# Effects of Reactive Species Produced by Electrolysis of Water Mist and Air through Non-Thermal Plasma on the Performance and Exhaust Gas of Gasoline Engines

**DOI:** 10.3390/molecules27207072

**Published:** 2022-10-20

**Authors:** Chia-Hsin Hsieh, Ming-Hsien Hsueh, Cheng-Wen Chang, Tao-Hsing Chen

**Affiliations:** 1 Department of Mechanical Engineering, National Kaohsiung University of Science and Technology, Kaohsiung 807618, Taiwan; 2 Department of Industrial Engineering and Management, National Kaohsiung University of Science and Technology, Kaohsiung 807618, Taiwan

**Keywords:** non-thermal plasma (NTP), water injection (WI), engine performance, exhaust emissions, brake-specific fuel consumption (BSFC), water electrolysis

## Abstract

Countries are paying increasing attention to environmental issues and are moving towards the goal of energy saving and carbon reduction. This research presents a method to analyse the effects of the use of non-thermal plasma (NTP) and water injection (WI) devices on the efficiency of internal combustion engines. The devices were installed on the intake manifold to investigate the effects of additional substances produced by electrolysis on the engine performance and exhaust emissions. According to the results, the addition of the NTP and WI devices affected the power efficiency and the rate of change of the brake-specific fuel consumption (BSFC) of the internal combustion engines. In addition, the change rate of hydrocarbons (HC), carbon monoxide (CO), and nitrogen oxides (NOx) in the exhaust gases was affected. In conclusion, the study found that the additional substances generated by the NTP-electrolysed water mist or air influenced the fuel combustion efficiency and exhaust emissions.

## 1. Introduction

Global energy demand has increased in the past decade, with an increase in the population. Energy supplies rely primarily on fossil fuels, including natural gas, coal, and petroleum, which support approximately 84% of the global primary energy demand [1]. Vehicles are one of the primary means of transportation for short-range human mobility. The internal combustion engine (ICE) is the main source of power for vehicles, and the energy of the power source is provided mainly by fossil-based source combustion. Fossil fuel combustion produces a combination of exhaust gas, thermal energy, and chemical energy. The contaminants in the exhaust gas include hydrocarbons (HC), nitrogen oxides (NOx), carbon monoxide (CO), carbon dioxide (CO_2_), and other gases [2,3]. At present, the rapid depletion of energy resources and the increasingly serious environmental pollution are the main issues of the world. Therefore, increasingly stringent regulations require engines to run cleaner [4,5]. Under the requirements of these regulations and fierce market competition, the main goal of the design was to find useful methods for low tailpipe emissions and low fuel consumption.

Water has long been considered a good engine coolant. Water injection (WI) in ICEs has been used since World War II and has a direct effect on the combustion and heat transfer processes [6]. It can economically reduce exhaust gas emissions while improving engine performance [7]. According to the location of the water injector, several different injection methods can be used, the cheapest and easiest of which is to inject water into the intake manifold [8]. BMW launched the M4 GTS model with direct fuel injection and WI in the intake manifold in 2015 [9]. Mingrui et al. (2017) investigated the influence of variable WI by mass on the performance and emission of a gasoline direct injection (GDI) engine under light load conditions; the results indicated that a 15% WI by mass used with fuel exhibited the best engine performance, and WI decreased the NOx emissions (ppm) and soot emissions [10]. Marchitto et al. (2018) investigated the effect of WI on fuel efficiency and particle number emissions in a turbo-charged SI engine. WI was found to have stopped mixture over-fuelling and improved fuel efficiency without engine load penalties. The higher knock tolerance and better combustion phasing allowed by the WI led to a significant reduction in particle emissions [11]. Min et al. (2019) investigated the effect of water vapour injection into the small compression ignition engine’s intake port on combustion and exhaust emission performance. The results showed that fuel consumption decreases with an increase in the amount of water vapour injection [12]. Zhuang et al. (2020) investigated the effect of adding a WI system to the intake manifold of a turbocharged gasoline direct-injection engine. The results showed that although WI has considerable potential for suppressing the engine knock, the ignition timing must be adjusted. If matched appropriately, not only can it effectively suppress an explosion earthquake, but it can also reduce NO and CO emissions in the engine exhaust [13]. Zhuang et al. (2020) investigated the water spray evolution process of port water injection (PWI) and its effect on engine performance by using the WI system. The results indicated that PWI can effectively suppress knocking and reduce the combustion temperature, and that operation at a stoichiometric air–fuel ratio with moderately advanced ignition timing will increase the thermal efficiency by nearly 6% [14]. Fratita et al. (2021) investigated the WI in spark ignition engines. The result showed that the mechanical work produced during expansion, in relation to the amount of fuel injected, is higher when WI is present. For a W/F ratio of more than 0.71, the researchers recorded a 4% mechanical work increase and a higher efficiency. However, the higher mechanical losses during intake, compression, and exhaustion decrease the overall efficiency of the indicated cycle; this trend can probably be inverted when the valve timing is optimised for these conditions of WI [15].

Non-thermal plasma (NTP) has been widely used in various industries. The plasma species are chemically active and can form new stable compounds. These reactive species can be used in various applications, such as assisting in combustion, surface modification, and wastewater treatment. Therefore, NTP systems are considered an important strategy in recovering exhaust pollutants. Adnan et al. (2017) investigated the effect of applying NTP to gasoline engine exhaust; the results showed that maintaining an appropriate spark gap and increasing the flow rate can effectively reduce the concentration of CO, CO_2_, HC, and NOx by 95% [16]. Wang et al. (2019) investigated the nitrogen oxide removal by NTP for marine diesel engines using a dielectric barrier discharge reactor to generate NTP for the exhaust denitration. The results indicated that in the NO/N_2_ system, the NTP removal efficiency of NO is close to 100%, and that the O_2_ concentration plays a decisive role in the denitration performance of the NTP. Upon the addition of NH_3_, the removal efficiency of NOx reaches up to 40.6% [17]. Sidik et al. (2019) investigated the removal of NOx from vehicles with diesel engines. They used the NTP to reduce the NOx from the diesel engine and compared the experimental and simulation results in terms of NOx reduction. The results indicated that reduction of NOx in the experiment and the simulation was 68.06% and 74.37%, respectively, indicating that the NTP provided effective treatment in terms of the oxidation process to remove NOx from vehicles with diesel engines [18]. Nur et al. (2019) investigated the removal of emission gases COx, NOx, and SOx from automobiles using NTP and used the corona discharge to reduce the emissions of gases released by the stationary-state diesel engine cars considered in the research. The results indicated that the best reduction level at 2200 rpm for COx was 86.52%, for CO was 88.93%, and for HC was 97.34%, and for NOx at 4600 rpm was 76.19% [19]. Hsueh et al. (2021) investigated the effect of using a water vapour injection system and the NTP system on the performance and emissions of the spark-ignition (SI) engine. The results showed that the installed water vapour system and NTP system on the intake manifold of the SI engine could reduce HC by approximately 16.31%, while 25 °C water + NTP and NOx could reduce it by approximately 11.88% [20].

Although many studies have explored the application of WI to ICEs, few studies have combined it with NTP electrolysis. The current study explored the effect of varying the engine speed and the air-fuel (A/F) ratio on the combustion efficiency by installing WI and NTP devices on the intake manifold. This study aimed to investigate the effect of the free radicals generated by NTP electrolysis on the engine combustion to analyse the engine’s performance and exhaust emissions. The result was directed towards providing a method to improve the air pollution issue caused by the SI engine application, in order to achieve environmental sustainability.

## 2. Experimental Apparatus and Techniques

### 2.1. Experimental Set-Up

**Figure 1:**
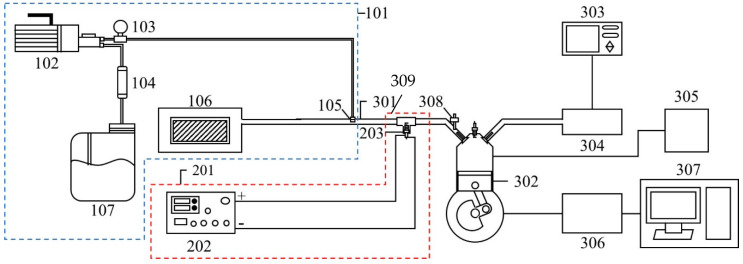
Schematic of the experiment and set-up location.

**Figure 2:**
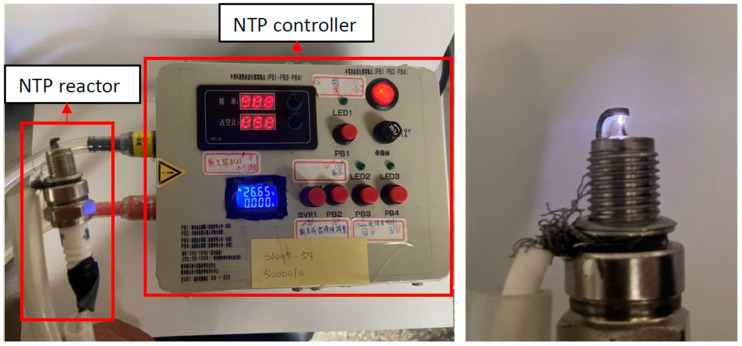
Photographic view of the device set-up on the NTP system.

**Figure 3:**
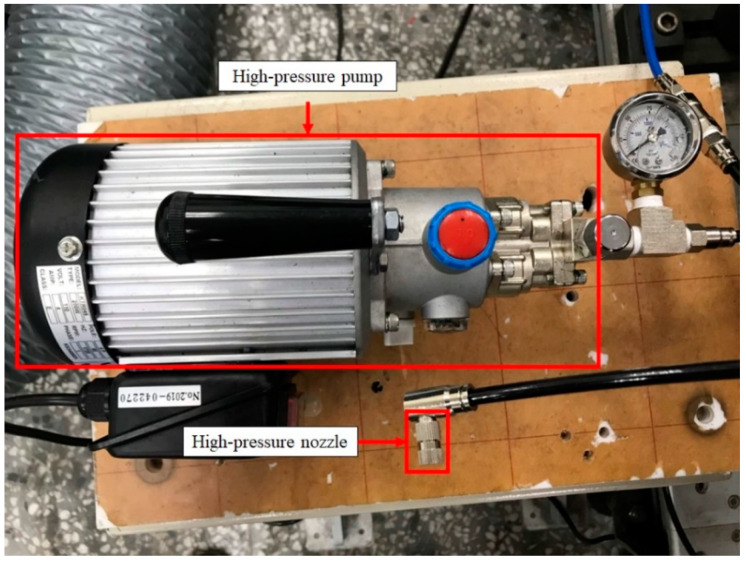
Photographic view of the device set-up on the WI system.

The experimental set-up of this study included the NTP system, the WI system, and experimental equipment. A schematic representation of the experiment is shown in Figure 1, and the detailed specifications of the experimental equipment are presented in Table 1. The NTP controller used in this experiment was the PDPS-5W model, manufactured by Creating Nano Technologies Co., Ltd. (Tainan City, Taiwan), and the CR7EIX reactor used was manufactured by NGK Spark Plug Co., Ltd. (Aichi Prefecture, Japan). The specifications of the NTP system are listed in Table 2 and the photographic view of the NTP system is shown in Figure 2. The physical diagram of the WI system is shown in detail in Figure 3, including the pump and the high-pressure nozzle. The WI system and the LV-119B pump used in the experiment were produced by the W3 Misting System Co., Ltd. (Tainan City, Taiwan). The WI system specifications are presented in Table 3. Furthermore, the experimental parameter settings of the NTP system and the WI system are listed in Table 4. The water consumption of the WI system in this experiment is maintained at 15 cc/min. During the experiment, the voltage of the NTP system was set at 10 V, the spark frequency at 100 Hz and 1000 Hz, and the duty cycle at 40%. Furthermore, the pressure of the WI system was set at 1000 psi, and a high-pressure nozzle with a diameter of 0.1 mm was used.

**Figure 4:**
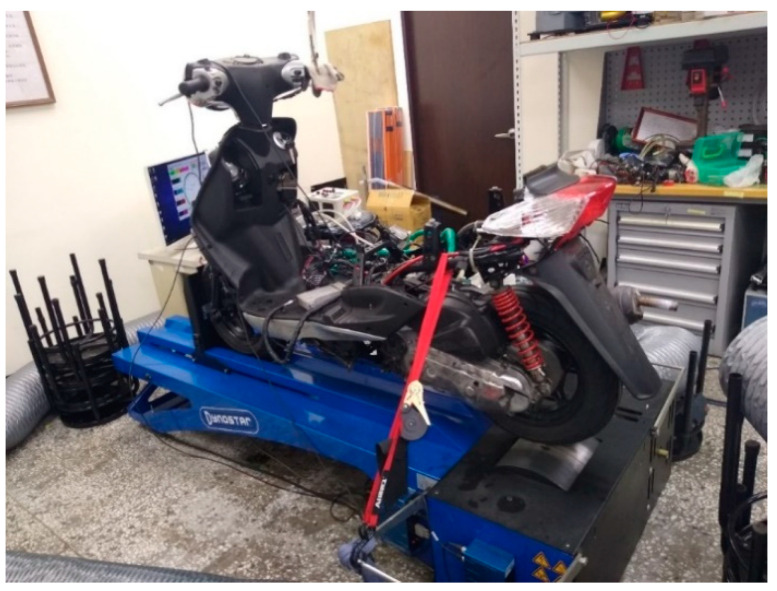
Photographic view of the device set-up on the experimental motorcycle.

The experimental equipment included an experimental motorcycle, an engine management system, an exhaust gas analyser, and a motorcycle chassis dynamometer. The experimental motorcycle used in the experiment was the Cygnus-X 125, manufactured by Yamaha Co., Ltd. (Hsinchu County, Taiwan), as shown in Figure 4. The engine specifications of the experimental motorcycle are listed in Table 5, and the control conditions of the engine are presented in Table 6. The engine management system is illustrated in Figure 4. The engine management system used in the experiment was the aRacer RC1 model, manufactured by aRacer SpeedTek Inc. (Hsinchu City, Taiwan). The engine management system was used to set up the A/F ratio of the experimental motorcycle from 10 to 14 for the experiment. This parameter was particularly set because the engine was easy to knock at less than 10, and the sparks were difficult to ignite with an A/F ratio of more than 14. Note that only a 100% load was used to make the air flow smoothly in the intake manifold.

The exhaust gas analyser used in the experiment was the EF-306EN exhaust gas analyser, manufactured by Exford Co., Ltd. (Taipei, Taiwan). The measurement accuracy of the exhaust gas analyser is presented in Table 7. The motorcycle chassis dynamometer used in the experiment was the D50ECB, manufactured by Dynostar Co., Ltd. (Taichung City, Taiwan); it was used to set the experimental motorcycle engine speed in the range of 4000–7000 rpm.

### 2.2. Reactions of NTP System

NTP electrolysed the mixture of the water mist and air to produce combustibles and combustion-supporting substances into the engine. The reaction formula of the water electrolysis by NTP was as follows [21]:

(1) $$e+H_{2}O\to e+\cdot H+\cdot \mathrm{OH}$$

(2) O(D1)+H2O→·OH+·OH

(3) $$\mathrm{OH}+\cdot \mathrm{OH}+M\to H_{2}O_{2}+M$$

(4) O(P3)+·OH→O2+·H

(5) $$O_{2}+\cdot H\to \cdot {\mathrm{HO}}_{2}$$

where O(D1) represents the excited-state oxygen atom and O(P3) denotes the ground-state oxygen atom.

High-energy electrons in the NTP collided and dissociated the gas molecules, such as N_2_ and O_2_ originally existing in the air, resulting in atoms and free radicals etc., after the air came into contact with the NTP. The reaction formula was as follows [21,22]:


(6) e+N2→e+N(S4)+N(S4)

(7) e+N2→e+N(D2)+N(D2)

(8) e+O2→e+O(P3)+O(P3)

(9) e+O2→e+O(P3)+O(D1)

(10) O(D1)+O2→O(P3)+O2

(11) O(D1)+N2→O(P3)+N2

(12) $$\cdot N+O_{2}\to \mathrm{NO}+\cdot O$$

(13) ·N+·O→NO

(14) $$\cdot O+{\mathrm{NO}}_{2}\to \mathrm{NO}+O_{2}$$

(15) $$\cdot N+{\mathrm{NO}}_{2}\to N_{2}+O_{2}$$

(16) $$\cdot N+\mathrm{NO}\to N_{2}+\cdot O$$

(17) $$\cdot O+{\;\mathrm{NO}}_{2}\to {\mathrm{NO}}_{3}$$

(18) e+NO→e+·O+·N

(19) $$e+{\;\mathrm{NO}}_{2}\to e+\mathrm{NO}+\cdot O$$

(20) $$\cdot O+O_{2}\to O_{3}$$

(21) $$\mathrm{NO}+O_{3}\to {\mathrm{NO}}_{2}+O_{2}$$

(22) $${\mathrm{NO}}_{2}+O_{3}\to {\mathrm{NO}}_{3}+O_{2}$$

where N(S4) represents the ground-state nitrogen atom and N(D2) denotes the excited-state nitrogen atom. Through the above reaction, free radicals or ions, such as $O_{3}$, ·O, and NO, were generated, which affected the combustion efficiency. According to the above description, water and air were electrolysed by NTP to produce different substances. Therefore, this paper discusses the effects of these substances on the performance and the exhaust emissions of gasoline engines.

### 2.3. Experimental Procedure

The main data recorded in this experiment included engine speed, horsepower, torque, air–fuel ratio, and exhaust emission (HC, CO, and NOx) concentration. In the experimental operation, the WI system was turned on or off according to the experimental requirements. The pressure was maintained at 1000 psi when the device was turned on. Note that the switch of the NTP system was required by the experiment. The A/F ratios in this experiment were set to 10, 11, 12, 13, and 14, and the rotational speeds were set to 4000, 5000, 6000, and 7000 rpm, respectively. The exhaust gas was measured by the exhaust gas analyser for 180 s after the value displayed by the computer application, connected to the chassis dynamometer, reached the required standard. Then, the data of the last 30 s were obtained for analysis. The measured speed and torque were used to calculate the horsepower value. The horsepower calculation formula used was as follows:

$$P=\frac{\left(T\times N\right)}{7123.6}$$

where P represents horsepower (hp), T denotes torque (N∙m), and N indicates engine speed (rpm). 

The calculation of the fuel consumption rate per unit of braking was based on the fuel injection pulse width displayed on the trip computer with the rotation speed, the nozzle flow rate, and the horsepower. The calculation formula used was as follows:

$$\mathrm{BSFC}=\left(I\times F\times \frac{N}{2}*60\right)/P\left(\mathrm{kW}\right)$$

where I represents injection pulse, F denotes nozzle flow (cc), and N refers to engine speed (rpm).

## 3. Results and Discussion

In this study, NTP and WI systems are installed. 100 Hz and 1000 Hz in WAN represented the start-up of NTP and WI systems. 0 Hz in WAN represented the start-up of only WI systems. 100 Hz and 1000 Hz in AN represented the start-up of only NTP systems.

### 3.1. Engine Performance

**Figure 5:**
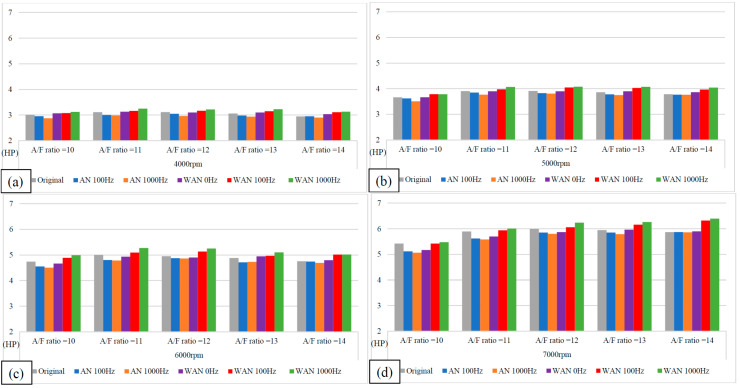
The horsepower values of WAN and AN with different frequencies at (**a**) 4000 rpm, (**b**) 5000 rpm, (**c**) 6000 rpm, and (**d**) 7000 rpm.

**Figure 6:**
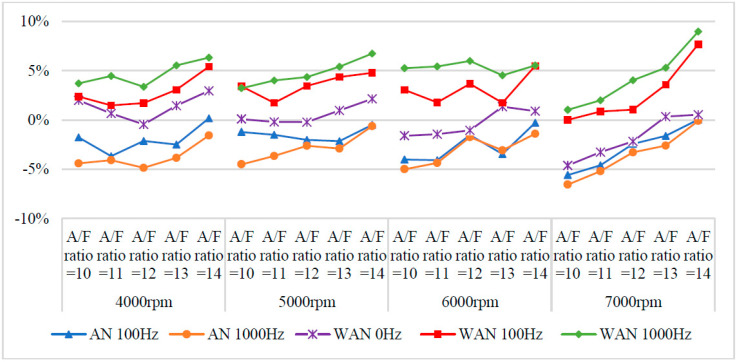
The horsepower change rate of WAN and AN with different frequencies.

Figure 5 and Figure 6 show the horsepower value and change rate of WAN and AN with different frequencies. The results from WAN 100 Hz and 1000 Hz demonstrated an increase in the horsepower change rate at various engine speeds and A/F ratios. This was because combustibles and combustion-supporting substances (such as H_2_ and O_2_) were produced by the NTP-electrolysed water mist to assist in engine combustion, improve the combustion efficiency, and increase the output of power performance [23]. In addition, the results at 1000 Hz were greater than those obtained at 100 Hz, which could be attributed to the increased possibility of the water mist being electrolysed into combustibles and combustion-supporting substances. The comparison of A/F ratio = 10 to 14 revealed that a higher A/F ratio caused a higher change rate of the horsepower. That is, the combustion efficiency of the fuel and the gas mixed with the combustibles and the combustion-supporting substances was higher under the condition of a high A/F ratio. However, the results of WAN 0 HZ demonstrated that the horsepower decreases as the engine speed increases, which is because the water reduces the engine temperature, and the combustion efficiency decreases. The decreased horsepower change rate in AN could be attributed to the relatively large negative effect of the free radicals generated by the electrolysis of air on the combustion reaction, which inhibited the fuel combustion and decreased the total power output energy [24,25]. In addition, the free radicals generated by air electrolysis will also reduce the volumetric efficiency of the engine, thereby reducing the engine torque [26]. The higher the frequency of NTP was, the greater the number of free radicals generated by the electrolysis of air was, resulting in the horsepower change rate at 1000 Hz being lower than that at 100 Hz.

### 3.2. BSFC Performance

**Figure 7:**
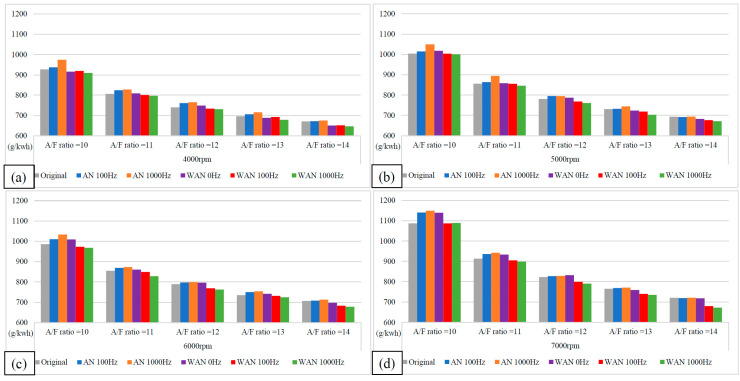
The BSFC values of WAN and AN with different frequencies at (**a**) 4000 rpm, (**b**) 5000 rpm, (**c**) 6000 rpm, and (**d**) 7000 rpm.

**Figure 8:**
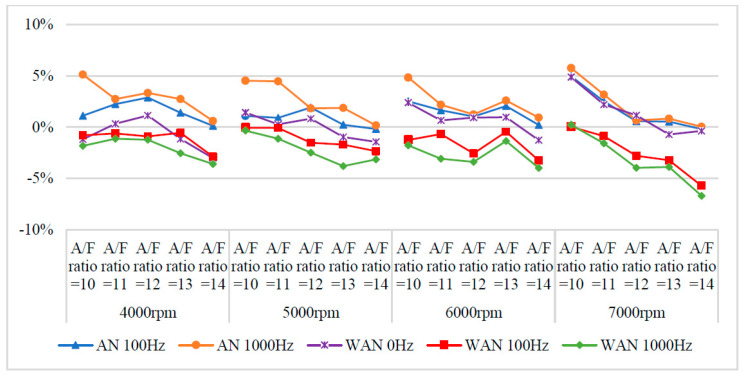
The BSFC change rate of WAN and AN with different frequencies.

Figure 7 and Figure 8 show the BSFC values and change rate of WAN and AN with different frequencies. The numerical value used in the calculation formula of BSFC in this experiment, as well as the nozzle flow rate and the rotation speed of the original fuel injector of the YAMAHA Cygnus-X 125, was calculated using the injection pulse width displayed in the computer program by the engine management system. The decreased BSFC change rate in WAN 100 Hz and 1000 Hz might be attributed to the faster combustion of hydrogen produced by the water electrolysis and the almost constant volume process [27]. The results obtained at 1000 Hz were lower than those obtained at 100 Hz, which could be attributed to the increase in the engine power when more combustibles and combustion-supporting substances were provided for the engine combustion. Water can reduce the combustion temperature and improve the latent heat of vaporisation of charge and specific heat capacity. Therefore, the BSFC change rate in WAN 0 Hz increases [28]. The increased BSFC change rate in AN was because of the large effect of free radicals causing the fuel to not burn to the limit, thereby reducing the combustion efficiency [24,25]. The higher the frequency of NTP was, the greater the number of free radicals generated by air electrolysis was and the higher the BSFC change rate was.

### 3.3. Exhaust Emissions

**Figure 9:**
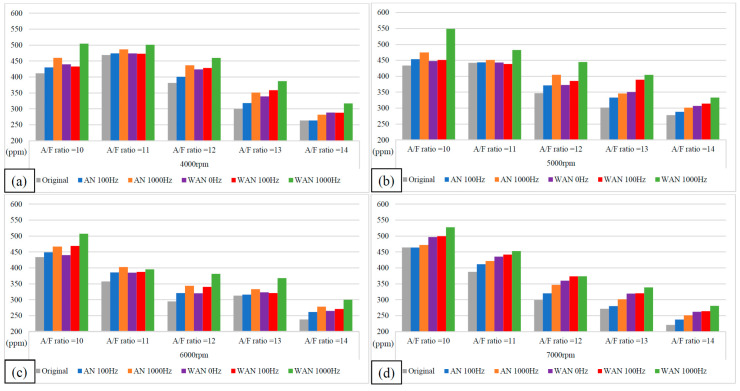
The HC values of WAN and AN with different frequencies at (**a**) 4000 rpm, (**b**) 5000 rpm, (**c**) 6000 rpm, and (**d**) 7000 rpm.

**Figure 10:**
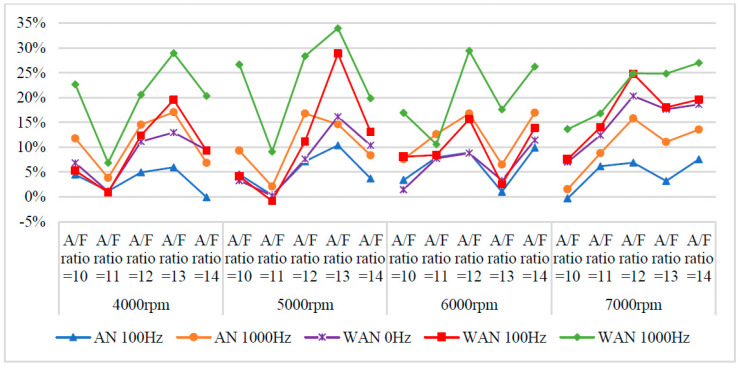
The HC change rate of WAN and AN with different frequencies.

Figure 9 and Figure 10 show the HC values and change rate of WAN and AN with different frequencies. The overall HC change rate had mostly increased. Note that the HC change rate of WAN increased the most, i.e., up to 34%. The increased HC change rate in WAN 100 Hz and 1000 Hz was attributed to the fact that the hydrogen produced by the electrolysis of water had a smaller amount of ignition energy than the fuel and was thus easier to combust. Part of the oxygen was also combusted with hydrogen, making it easier for the fuel to be discharged with incomplete combustion [29]. In addition, the results obtained at 1000 Hz were higher than those obtained at 100 Hz because more hydrogen was produced by the water electrolysis, resulting in more fuel being emitted with incomplete combustion. The BSFC change rate in WAN 0 Hz increases because the water reduces the engine temperature, and the combustion efficiency decreases. The increased HC change rate in AN was attributed to the fact that the free radicals generated by the air electrolysis inhibited the fuel combustion, resulting in a part of the fuel being emitted with incomplete combustion [24]. Moreover, a part of the oxygen was combined with nitrogen during the NTP electrolysis, which reduced the amount of oxygen entering the engine, resulting in the fuel being emitted with incomplete combustion.

**Figure 11:**
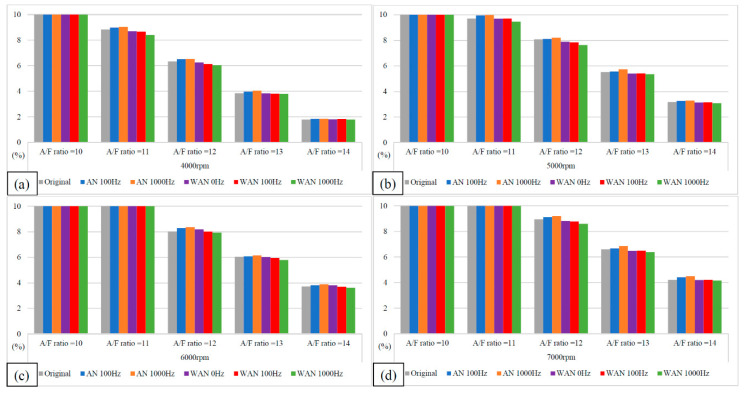
The CO values of WAN and AN with different frequencies at (**a**) 4000 rpm, (**b**) 5000 rpm, (**c**) 6000 rpm, and (**d**) 7000 rpm.

**Figure 12:**
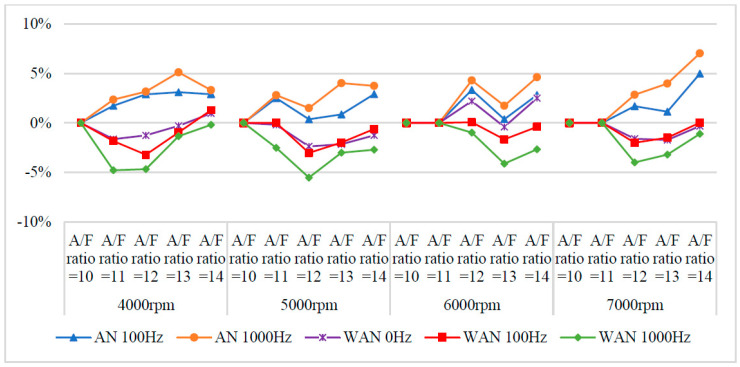
The CO change rate of WAN and AN with different frequencies.

Figure 11 and Figure 12 show the CO values and change rate of WAN and AN with different frequencies. The maximum decrease in the CO change rate was observed in the case of WAN because electrolysis of water produces hydrogen, which does not contain any carbon in its structure and thus does not produce CO when combusted with oxygen. However, the combustion of part of the oxygen and hydrogen leads to an incomplete combustion of the fuel, resulting in an increase in CO because of insufficient oxygen during the combustion process of the fuel [28]. In addition, the result obtained at 1000 Hz was lower than that obtained at 100 Hz because the more hydrogen produced by water electrolysis burned lower CO emissions. The increased CO change rate in AN was because a part of the oxygen in the intake air combined with nitrogen to pass through the NTP system, resulting in an incomplete combustion of the fuel in the engine [30]. Furthermore, more oxygen and nitrogen combined through the higher NTP electrolysis frequency, resulting in higher CO values.

**Figure 13:**
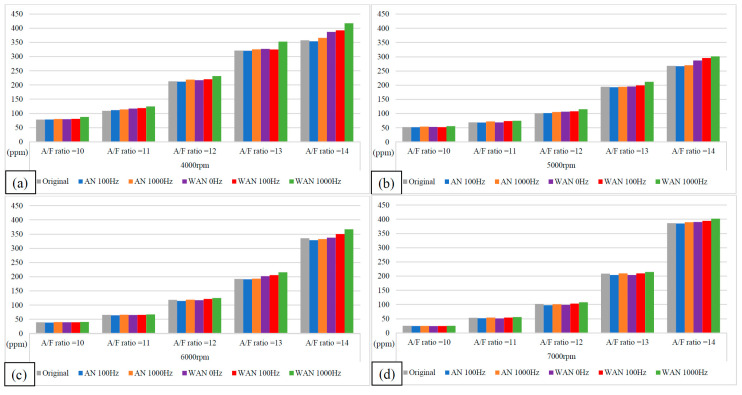
The NOx values and change rate of WAN and AN with different frequencies at (**a**) 4000 rpm, (**b**) 5000 rpm, (**c**) 6000 rpm, and (**d**) 7000 rpm.

**Figure 14:**
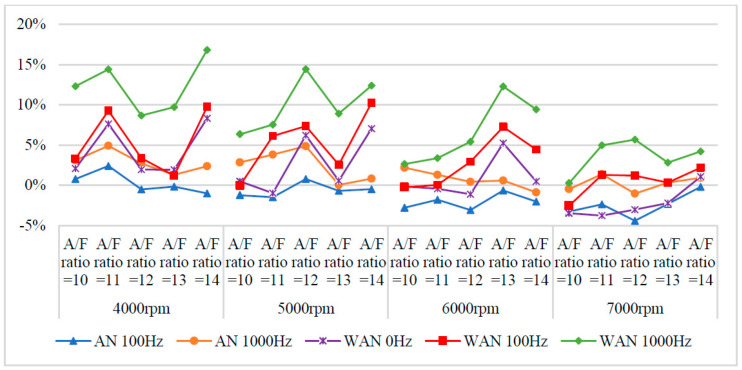
The NOx change rate of WAN and AN with different frequencies.

Figure 13 and Figure 14 show the NOx values and change rate of WAN and AN with different frequencies. The increased NOx change rate in WAN 100 Hz and 1000 Hz was because the hydrogen and oxygen produced by the water electrolysis helped the engine combustion, resulting in an increase in the combustion temperature. The nitrogen and oxygen were easily recombined to form NOx at high temperatures [31]. In addition, the slight decrease in the NOx change rate at 6000 rpm and 7000 rpm was attributed to the larger amount of water mist entering the combustion chamber, which reduced the thermal efficiency of the combustion chamber and the temperature. The results obtained at 1000 Hz were higher than those obtained at 100 Hz because the water electrolysis produced more hydrogen and oxygen to aid in the combustion, resulting in higher combustion temperatures. The results of WAN 0 HZ demonstrated that the NOx decreases as the engine speed increases, which is because the water reduces the engine temperature. The increased NOx change rate in AN might be attributed to the recombination of nitrogen and oxygen into NO during the air electrolysis [22]. However, the free radicals generated by the air electrolysis cause incomplete combustion of the fuel, and reducing the combustion temperature decreased the NOx change rate. 

## 4. Conclusions

In this study, the WI and NTP devices installed on the intake manifold were experimentally investigated, and the water mist and air were electrolysed through the NTP reactor to generate additional substances to explore the effect on the gasoline engine. These devices increased horsepower, HC, and NOx in the presence of WI and NTP (WAN 100 Hz and 1000 Hz) and reduced BSFC and CO. The BSFC, HC, and NOx were mostly increased in NTP broken (WAN 0 Hz), and they mostly reduced CO. The BSFC, HC, CO, and NOx were mostly increased in the absence of water (AN), and they reduced the horsepower. The magnitude of the change increased with an increase in the NTP frequency irrespective of WAN or AN. In conclusion, the horsepower, BSFC, and CO improved upon the addition of WI and NTP (WAN 100 Hz and 1000 Hz), but HC and NOx emissions decreased. The horsepower, BSFC, HC, CO, and NOx decreased after the water ran out (AN), and the higher the frequency of NTP was, the more severe it was. Therefore, the research reported in this paper will provide a valuable reference for the application and development of NTP and WI systems to improve engine performance, save energy, and reduce CO emissions.

## Figures and Tables

**Table 1:** Detailed equipment name of the experimental equipment.

101	WI system	301	Intake manifold
102	Pump	302	SI engine
103	Pressure gauge	303	Exhaust gas analyser
104	Water filter	304	Exhaust pipe
105	High-pressure nozzle	305	Engine management system
106	Air filter	306	Motorcycle chassis dynamometer
107	Bucket	307	Computer
201	NTP system	308	Fuel injector nozzle
202	NTP controller	309	Intake valve
203	NTP reactor		

**Table 2:** Specification of NTP system.

NTP controller	Voltage (V)	0~32
	Frequency (Hz)	1~1000
	Duty cycle (%)	0~100
NTP reactor	Gap (mm)	0.75
	Centre electrode diameter (mm)	0.6
	Outer electrode diameter (mm)	1.85

**Table 3:** Specification of WI system.

Pump	Flow of water (L/M)	0~1.2
	Pressure of water (PSI)	0~1000
High-pressure nozzle	Nozzle hole diameter (mm)	0.1
	Water spray diameter (µm)	8~10

**Table 4:** Control conditions of the NTP system & WI system.

NTP system	NTP controller	Voltage (V)	10
		Frequency (Hz)	100, 1000
		Duty cycle (%)	40
WI system	Pump	Pressure of water (PSI)	1000
		Water temperature (°C)	25
		Water consumption (cc/min)	15

**Table 5:** Engine specifications of the motorcycle.

Motorcycle Name & Type	Cygnus-X 125
Engine type	4 stroke SOHC
Cylinder arrangement	Single cylinder
Cooling type	Forced air-cooled
Curb Weight (kg)	119
Wheelbase (mm)	1295
Fuel	95 # Unleaded gasoline
Displacement (cc)	124
Bore × Stroke (mm)	52.4 × 57.9
Compression ratio	10:1
Max power (kW/rpm)	7.8/8500
Max torque (N-m/rpm)	9.2/7500

**Table 6:** Control conditions of the engine.

Engine speed (rpm)		4000, 5000, 6000, 7000
Load (%)		100
Fuel octane rating		95
Engine temperature (oC)		75–80
Intake air temperature (oC)		22–26
Tyre pressure (PSI)	Front wheel	27
	Rear wheel	30

**Table 7:** Measurement accuracy of the exhaust gas analyser.

Gas	Measurement Range	Absolute Accuracy
HC	0~2000 ppm	±12 ppm
CO	0~10%	±0.06%
CO_2_	0~20%	±0.5%
O_2_	0~25%	±0.1%
NOx	0~5000 ppm	±25 ppm

## Data Availability

Not applicable.

## Sample Availability

Samples of the compounds are available from the authors.
